# Comparative and phylogenetic analysis of chloroplast genomes in the subtribe Leptoboeinae (Gesneriaceae)

**DOI:** 10.3389/fpls.2026.1766257

**Published:** 2026-03-23

**Authors:** Zibing Xin, Xiaomao Qian, Xiaojuan Li, Longfei Fu, Xinxiang Bai, Fang Wen

**Affiliations:** 1 Guangxi Key Laboratory of Plant Conservation and Restoration Ecology in Karst Terrain, Guangxi Institute of Botany, Guangxi Zhuang Autonomous Region and Chinese Academy of Sciences, Guilin, Guangxi, China; 2 Gesneriad Committee of China Wild Plant Conservation Association, National Gesneriaceae Germplasm Resources Bank of GXIB, The Gesneriad Conservation Center of China, Guilin Botanical Garden, Chinese Academy of Sciences, Guilin, Guangxi, China; 3 College of Forestry, Guizhou University, Guiyang, Guizhou, China

**Keywords:** comparative chloroplast genomics, Leptoboeinae, molecular markers, phylogenetic relationships, polyphyly

## Abstract

The subtribe Leptoboeinae (Gesneriaceae: Trichosporeae) comprises seven genera—*Rhynchotechum* Blume, *Boeica* C.B.Clarke, *Beccarinda* Kuntze, *Leptoboea* Benth., *Platystemma* Wall., *Crassicaulis* Lei Cai & Mich.Moller, and *Championia* Gardner. It is highly valuable in horticulture and medicine. However, long-standing taxonomic issues, including the polyphyly of *Boeica* and the uncertain placement of *Championia*, remain unresolved. To address these problems, we sequenced and assembled complete chloroplast genomes for 17 species representing all seven genera, and conducted comparative genomic and phylogenomic analyses. The genomes ranged from 153,114 to 154,647 bp and exhibited a typical quadripartite structure consistent with other Gesneriaceae. A total of 1,861 SSRs, 1,179 dispersed repeats, and 29 high-frequency codons with similar usage patterns were identified. We also detected 10 highly variable regions that may serve as potential molecular markers. the plastid tree and coalescent tree constructed based on CDS sequences in this study showed highly congruent topologies at the generic level, supporting the division of *Boeica* into five clades, two of which can be distinguished morphologically. *Championia* formed a sister relationship with all sampled genera of Trichosporeae and Epithemateae, confirming that it does not belong to Leptoboeinae. Moreover, structural variation in the plastome helped clarify the polyphyly of *Boeica*: the five clades exhibited significant differences in total GC content, LSC GC content, and SSC GC content. Given the overlapping geographic distributions of these clades, we infer that plastome divergence may reflect independent lineage evolution. Overall, this study offers new insights into the phylogenetic relationships within Leptoboeinae and provides a foundation for future taxonomic revision and evolutionary research.

## 1 Introduction

The subtribe Leptoboeinae was established in 1884 by Clarke, based on the characteristics that its capsules dehisce into 2 valves septicidally and its seeds have no appendages (Clarke, 1884). Later, Fritsch (Fritsch, 1894) merged the genera *Championia*, *Platystemma*, *Boeica*, and *Leptoboea* into the Championieae on the basis of their narrowly elongate calyx, 4 fertile stamens, and dehiscent fruits, and later transferred them to the Trichosporeae. At present, phylogenetic relationships within *Primulina* Hance and *Petrocodon* Hance (Trichosporeae) are relatively clear (Gu et al., 2024; Hsieh et al., 2022). However, members of Leptoboeinae are scattered across multiple countries along the southern slopes of the Himalayas, southern China, the Indochinese Peninsula, and two countries in the Indian subcontinent (Wang et al., 1990; POWO, 2025). The difficulty of collecting representative samples has long hindered research on this subtribe, to the extent that it has been largely overlooked, whether deliberately or unintentionally. In modern classification of Gesneriaceae, Leptoboeinae is treated as a subtribe of Trichosporeae and comprises seven genera—*Radiaticorollarus*, *Rhynchotechum*, *Boeica*, *Beccarinda*, *Leptoboea*, *Platystemma*, *Championia*, and *Crassicaulis* (Weber et al., 2013; Yang et al., 2025), encompassing approximately 48 species, distributed mainly in southern and southwestern China (Wang et al., 1990, 1998; Weber et al., 2013; Wen et al., 2022), and elsewhere in the above-mentioned regions. Members of this subtribe show striking morphological diversification and conspicuous differentiation, but they share relatively consistent traits such as an inconspicuous or capitate stigma, capsules dehiscing loculicidally into 2 valves, and 4 stamens (Weber et al., 2013), which distinguish them from other subtribes. Some genera of Leptoboeinae are traditionally used in folk medicine. For instance, many species of *Rhynchotechum* and *Boeica fulva* C. B. Clarke have medicinal or even edible value. For example, *Rhynchotechum vestitum* Wall. ex C. B. Clarke can be used in the treatment of hepatitis A and hepatitis B (Lu et al., 1998). At the generic level, the placement of *Championia* has remained controversial. Such as in subtribe Didymocarpeae of tribe Cyrtandreae by Bentham (1876); subtribe Championiinae of tribe Championieae by Fritsch 1894; later it was reassigned by different researchers to the tribe Didymocarpeae of subfamily Cyrtandroideae (Burtt, 1963; Ivanina, 1965; Burtt and Wiehler, 1995). Weber et al. (2013) on the basis of the tetramerous corolla, suggested its possible affinity with Leptoboeinae, but molecular phylogenetic evidence was lacking. Ranasinghe et al. (2024) using phylogenies based on *ndhF*, *matK*, *rps16*, and *trnL-F* sequences, found *Championia* to fall outside the Leptoboeinae clade. Moreover, plastome phylogenies based on protein-coding genes and coding regions indicated that *Radiaticorollarus* forms a sister group with some species of *Boeica and Rhynchotechum* (Wen et al., 2022), whereas whole plastome data (excluding one inverted repeat) supported *Radiaticorollarus* as sister to *Leptoboea* (Cui et al., 2023). However, neither relationship is well supported, resulting in continued uncertainty over its circumscription. Accordingly, Weber et al. (2025) after re-evaluating the morphological characteristics and phylogenetic relationships of *Radiaticorollarus*, treated it as part of the genus *Boeica*. *Boeica* itself was established by Clarke in 1874 on the basis of floral traits, including an almost actinomorphic corolla, four free stamens, and cylindrical capsules dehiscing dorsally along both locules. Several studies have shown that *Boeica* is not monophyletic, with some of its species forming a sister group to *Rhynchotechum* (Yang et al., 2020; Wen et al., 2022; Yang et al., 2023a), but Weber et al. (2025) argued that the sampling of *Boeica* and *Rhynchotechum* in the studies by Yang et al. (2020; 2023a) and Wen et al. (2022) was insufficient, and thus their results require further verification (Middleton et al., 2015), using ITS and *trnL-F*, also found *Boeica* and *Rhynchotechum* to be closely related and noted that several species of both genera exhibit alternating phyllotaxy with opposite leaves. Furthermore, Yang et al. (2025) reconstructed phylogenetic trees using plastid genome and low-copy nuclear (LCN) gene datasets, which revealed a complex reticulate evolutionary history within the subtribe and confirmed the polyphyly of *Boeica*, although no further analysis of this polyphyly was conducted.

With the rapid rise of next-generation DNA sequencing (NGS) technologies, the cost of sequencing has dropped dramatically, driving the swift advancement of genomics and enabling the application of large-scale molecular datasets in phylogenetic research, thereby giving rise to the field of phylogenomics (Sun et al., 2022; Wang et al., 2024a; Torruella et al., 2025). Chloroplasts are ubiquitous organelles in plants, and chloroplast genomes, due to their structural stability, moderate evolutionary rate, sequence conservation, good collinearity, and predominantly maternal inheritance, have become highly valuable in phylogenetic studies. They not only enhance the support and resolution of phylogenetic trees but also play an important role in elucidating species evolution and interspecific relationships (Zhai et al., 2021; Wu et al., 2024; Cheng et al., 2025; Dong et al., 2025; Xia et al., 2025).

Therefore, in response to the above issues and current situation, the main objectives of this study are: (1) to construct a phylogenetic framework of Leptoboeinae based on different chloroplast genome datasets, investigate conflicts between plastid trees and coalescent trees, and elucidate the phylogenetic relationships among genera within the subtribe; (2) to identify structural variations and potential phylogenetic signals through comparative analyses of chloroplast genomes, in order to clarify the polyphyly of *Boeica*; and (3) to detect highly variable regions of the chloroplast genome in Leptoboeinae and screen for potential DNA barcode markers.

## 2 Materials and methods

### 2.1 Plant materials and DNA extraction

In this study, we conducted species-level sampling of all known genera within the subtribe Leptoboeinae, collecting 17 species representing 7 genera (**Supplementary Table 2**), thereby covering all genera of the subtribe. To enhance dataset completeness, the 17 newly sequenced chloroplast genomes were integrated with previously published chloroplast genome data from 69 accessions of Leptoboeinae (Wen et al., 2022; Yang et al., 2025). This resulted in a final dataset of 86 chloroplast genomes representing 37 species (77.08% of the subtribe). The sampling included 1 species of *Championia* (100% of the genus), 1 species of *Platystemma* (100% of the genus), 1 species of *Crassicaulis* (100% of the genus), 8 species of *Beccarinda* (75% of the genus), 13 species of *Boeica* (76.47% of the genus), 2 species of *Leptoboea* (50% of the genus), 11 species of *Rhynchotechum* (61.11% of the genus), Among these, 37 representative samples were selected for chloroplast genomes structural analyses. Detailed information is provided in **Supplementary Table 2**.

Total genomic DNA of each sample was extracted using the modified CTAB method (Doyle and Doyle, 1987). A 1% agarose gel was prepared, and electrophoresis was performed to assess the quality of the extracted DNA, while DNA concentration and purity were measured using a Nanodrop spectrophotometer. Qualified samples were then sent to Sangon Biotech (Shanghai) Co., Ltd. for genomic library construction and high-throughput sequencing.

### 2.2 Sequencing, assembly and annotation

DNA libraries were sequenced on the Illumina high-throughput platform using paired-end 150 bp (PE) sequencing, generating 4 GB of raw data for each sample. The quality of the raw reads was assessed using FASTQ 0.36 (Chen et al., 2018), and adapters as well as low-quality reads were filtered with Trimmomatic v0.39 (Bolger et al., 2024) to obtain high-quality data (clean reads). Clean reads were assembled with GetOrganelle v1.7.5 (Jin et al., 2020) using the parameters “-R 20 -k 21, 45, 65, 85, 105, 127 -F embplant_pt.” Redundant contigs were manually removed with Bandage to obtain complete chloroplast genomes. Using *Beccarinda tonkinensis* (Pellegr.) Burtt (GenBank: NC_072338) as the reference, 30 chloroplast genome sequences were annotated with the GeSeq online annotation tool (Tillich et al., 2017). Coding sequence (CDS) start and stop codons were manually checked and adjusted in Geneious 9.0.2 (Kearse et al., 2012), while the type, length, and anticodon of tRNA genes were verified and corrected with tRNAscan SE (Chan and Lowe, 2019). The annotated information was visualized with OGDRAW v.1.3.163 (Greiner et al., 2019). To assemble the nrDNA repeat sequences from Illumina reads, GetOrganelle v1.7.5 (Jin et al., 2020) was also used with default settings and the parameters “-R 20 -k 21, 45, 65, 85, 105, 127 -F embplant_nr,” resulting in contigs for each sample. These contigs were then imported into Geneious 9.0.2 and aligned against the nrDNA reference sequence of *Beccarinda tonkinensis* (GenBank: KJ475423) for validation.

### 2.3 Chloroplast genome IR/SC boundary analysis, similarity comparison, and identification of highly variable regions

The boundaries of the 37 chloroplast genomes were compared and visualized using CPJSdraw (Li et al., 2023). To identify and visually detect interspecific variation regions in the chloroplast genomes, the online software mVISTA (https://genome.lbl.gov/vista/mvista/submit.shtml) (Frazer et al., 2004) was employed, with the annotation of *Beccarinda argentea* XZB2310 as the reference and LAGAN selected as the alignment program, to generate visual similarity comparisons of the 37 chloroplast genomes. Furthermore, DnaSP v.6.12.03 (Rozas et al., 2017) was used to analyze the aligned sequence matrix of the 37 chloroplast genomes in order to further quantify nucleotide polymorphism levels and detect highly variable regions within the subtribe Leptoboeinae. The sliding window analysis was performed with a window length of 800 bp and a step size of 200 bp, and the top 5% of windows with the highest *Pi* values were considered mutational hotspot regions (Wang et al., 2022; Gu et al., 2024).

### 2.4 Codon usage bias analysis

To quantify codon usage bias, CodonW 1.4.2 (Peden, 1999) was used to analyze and filter 37 CDS sequences. Since short sequences cannot accurately calculate the effective number of codons (Liu, 2013), only CDS longer than 300 bp were selected for Relative Synonymous Codon Usage (RSCU) analysis in this study. RSCU represents the ratio between the observed frequency of a codon and its expected frequency under equal usage (Sharp and Li, 1987). When RSCU > 1, the codon is used more frequently and considered a high-frequency codon; when RSCU < 1, the codon is used less frequently and considered a low-frequency codon; and when RSCU = 1, the codon shows no usage bias (Mazumdar et al., 2017).

### 2.5 Repeat sequence analysis

Simple sequence repeats (SSRs) were detected using Krait (Du et al., 2018) with the following parameters: mononucleotide repeat units ≥ 10, dinucleotide repeat units ≥ 5, trinucleotide repeat units ≥ 4, and tetra-, penta-, and hexanucleotide repeat units ≥ 3. Dispersed repeats, which are distributed throughout the genome, include 4 types: forward (F), reverse (R), palindromic (P), and complement (C) repeats (Jelinek et al., 1980). Dispersed repeats were identified using the online REPuter software (Kurtz et al., 2001), with a minimum repeat size of 30 bp and a sequence identity threshold of 90% (Hamming distance = 3).

### 2.6 Phylogenetic analysis

To investigate the systematic issues within the subtribe Leptoboinae, including intergeneric relationships and generic delimitation, we conducted phylogenetic analyses based on 103 chloroplast genomes and 107 nrDNA sequences. The chloroplast dataset included 37 species (86 samples) of Leptoboinae as the ingroup and 17 species from the closely related tribes Trichosporeae, Epithemateae, and Gesnerieae, as well as Scrophulariaceae and Lamiaceae, as outgroups. The nrDNA dataset comprised 37 species (86 samples) of Leptoboinae and 21 outgroup species corresponding to those used in the chloroplast dataset. These outgroups were selected because they are known to share close phylogenetic affinities with Leptoboinae, providing a robust framework for the analyses (Ranasinghe et al., 2024), while species from Scrophulariaceae and Lamiaceae were included to root the phylogenetic trees (Li et al., 2021). Four datasets were employed for phylogenetic analyses: (a) the nrDNA region 18S–ITS1–5.8S–ITS2–26S, (b) 81 concatenated CDS derived from 37 chloroplast genomes, (c) 81 CDS analyzed using a coalescent-based approach. The concatenation method was used based on the traditional view that chloroplast genomes evolve as a single unit, which was thought to avoid conflicts between gene trees and species trees (Doyle, 1992; Wicke et al., 2011), and (d) complete chloroplast genome sequences (with one IR region removed). However, recent studies have shown inconsistencies between gene trees and species trees constructed from chloroplast genomes (Walker et al., 2019; Xue et al., 2024). Therefore, this study used concatenation and coalescent methods based on chloroplast genome data to detect any structural inconsistencies. Phylogenetic trees were reconstructed using Bayesian inference (BI) and maximum likelihood (ML) methods. Multiple sequence alignments of the chloroplast genomes and nrDNA data were performed with MAFFT v.7.5.1.1 (Katoh and Standley, 2013) using default parameters. The alignments were manually checked and corrected in MEGA 11.0.13 (Tamura et al., 2021) to obtain final alignment matrices. DAMBE v5.3.19 (Xia, 2013) was used to evaluate the substitution saturation index (Iss) of the data matrices, with results indicating that the observed index of substitution saturation (Iss = 0.0800) was significantly lower than the critical Iss.c value (0.8610; P = 0.0000 < 0.05), suggesting that the data were not saturated and were suitable for phylogenetic tree reconstruction.

The optimal substitution model for both maximum likelihood (ML) analysis and Bayesian inference (BI) was determined using PhyloSuite v.1.2.3 (Zhang et al., 2020) based on the AICc criterion, with GTR+I+G identified as the best-fit model. ML and BI phylogenetic trees were constructed using IQ-TREE 1.6.12 and MrBayes 3.2.7, respectively, via PhyloSuite v.1.2.3. Branch support in the ML analysis was assessed using standard 1,000 bootstrap replicates (BS). BI inference was run for 1,000,000 generations of MCMC, sampling every 1,000 generations, and convergence was verified with ESS > 200. After discarding the first 25% of samples as burn-in, posterior probabilities (PP) were used to evaluate branch reliability. Single-gene trees were inferred in IQ-TREE 1.6.12 with 1,000 bootstrap replicates and automatic model selection. Coalescent tree inference was performed in ASTRAL-III v5.7.8 (Zhang et al., 2018), with branch support evaluated using local posterior probabilities (LPP; Sayyari and Mirarab, 2016) and quartet scores. Quartet frequencies were used to detect gene tree conflicts, reflecting the degree of incongruence among gene trees. Bootstrap values from gene trees were mapped onto the ASTRAL topology, and phypartspiecharts.py was used to generate pie charts visualizing the proportion of gene trees supporting each topology. Phylogenetic trees were visualized using Figtree v.1.4.4 (https://github.com/rambaut/figtree/releases, accessed 18 January 2025) and iTOL (Letunic and Bork, 2021).

### 2.7 Comparative analysis of chloroplast genomes

**Figure 1:**
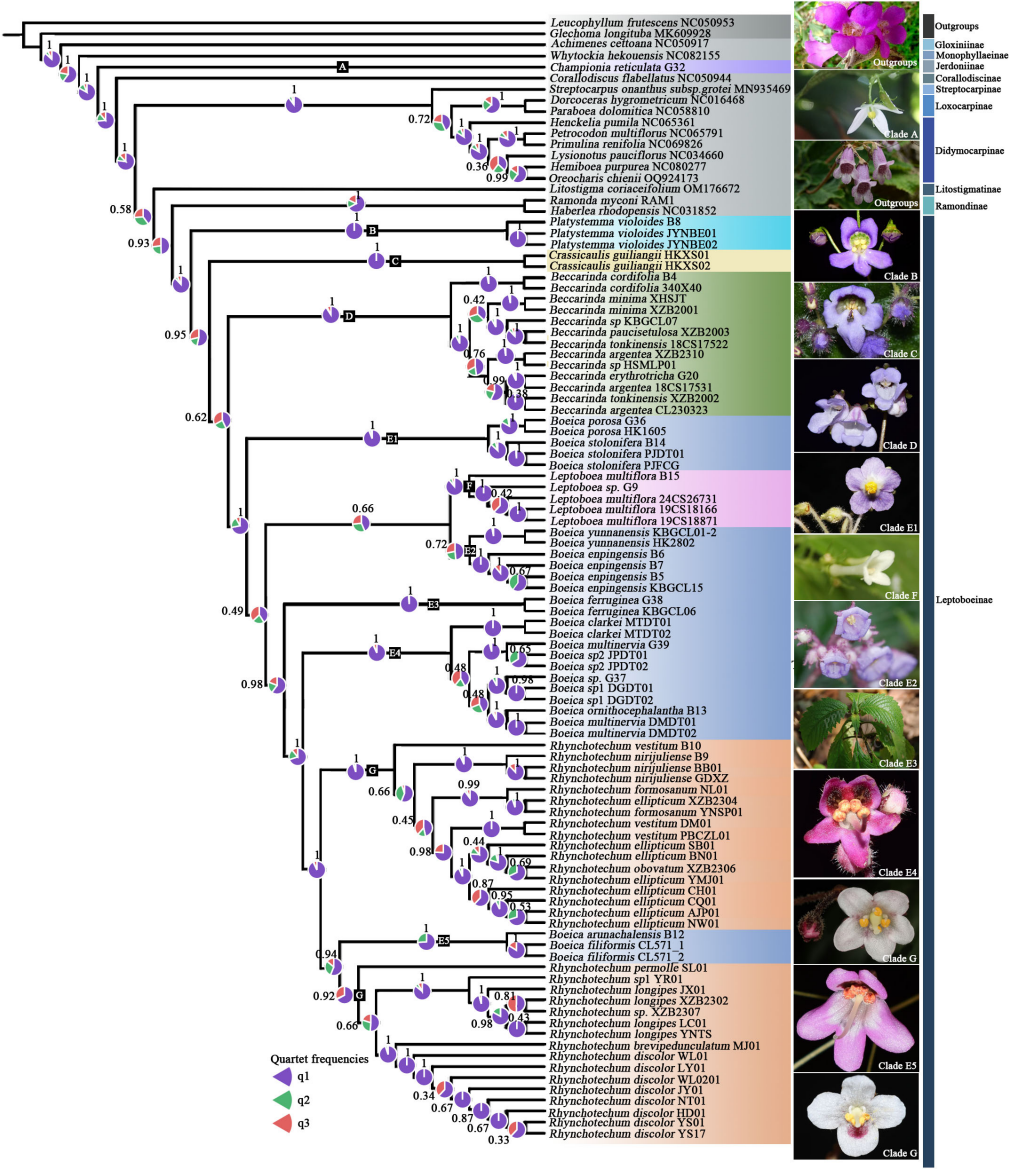
Coalescent tree: phylogenetic relationships of the subtribe Leptoboeinae inferred from 81 CDSs, with branch values indicating local posterior probability (LPP) support; pie charts around branches show the relative frequencies of three alternative topologies (purple, consistent with the coalescent tree; orange, first alternative topology; green, second alternative topology).

Samples used for chloroplast comparative genomic analyses were grouped according to the clades defined in **Figure 1**: E1 (2 species, 5 samples), E2 (2 species, 8 samples), E3 (1 species, 2 samples), E4 (6 species, 12 samples), and E5 (2 species, 3 samples). The chloroplast genome size, LSC, SSC, IR regions, and their corresponding GC contents were summarized for each clade and visualized using EcoAmp v0.13. We further compiled these genomic characteristics for all clades (A–G) of Leptoboeinae and generated boxplots in R v3.6.1. One-way ANOVA was performed in IBM SPSS Statistics v27, followed by Tamhane’s T2 *post-hoc* test without assuming equal variances, to assess significant differences among genera and subclades. Details and results are provided in **Supplementary Figure 3** and **Supplementary Table 6**.

## 3 Result

### 3.1 Structure and characteristics of chloroplast genomes

**Figure 2:**
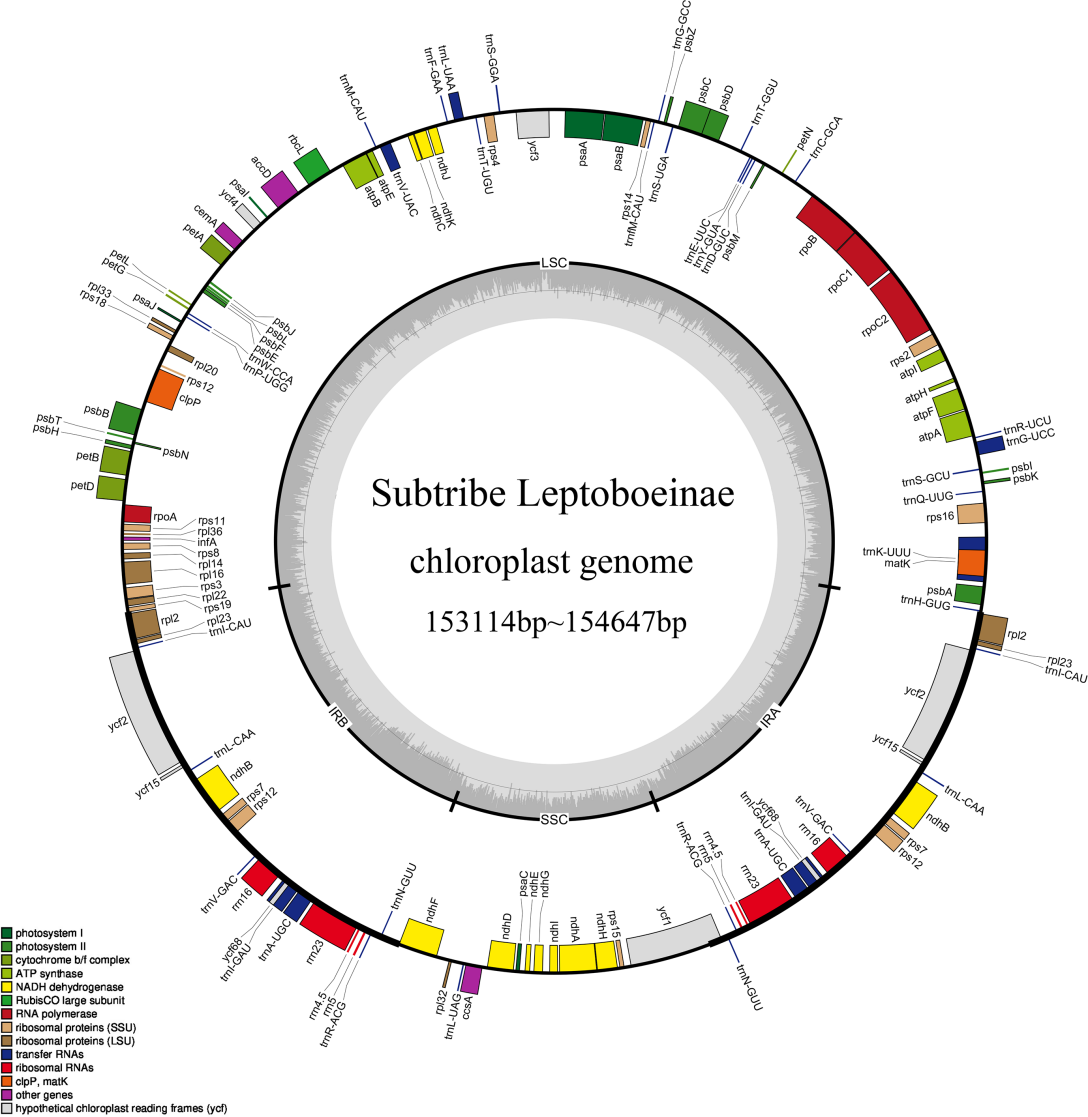
Gene map of 37 Species in the subtribe Leptoboeinae. Genes outside the circle are transcribed clockwise, and genes inside the circle are transcribed counterclockwise. *Rhynchotechum* was used as the template. Other species are shown in **Supplementary Figure 1**.

In this study, the chloroplast genomes of 37 species of Leptoboeinae were characterized, of which 17 were newly generated (**Supplementary Tables 1**, **2**). The total length of the chloroplast genomes ranges from 153,114 bp (*Platystemma violoides* Wall., B8) to 154,647 bp (*Rhynchotechum nirijuliense* Taram & D. Borah, B9), exhibiting a typical quadripartite circular structure (**Figure 2**). Each genome consisted of a large single-copy region (LSC) of 84,457–85,796 bp, a small single-copy region (SSC) of 17,691–19,569 bp, and two inverted repeats (IRs) of 24,635–25,534 bp. The overall GC content of the 37 chloroplast genomes was similar (**Supplementary Table 1**), ranging from 37.28% to 37.9%. The GC contents of the LSC and SSC regions were 35.17%–35.94% and 31.06%–31.94%, respectively, whereas that of the IR regions was higher, at 43.22%–43.29%. For protein-coding sequences (CDSs), the total GC content ranged from 37.28% to 37.9%, with codon positions 1, 2, and 3 (GC1, GC2, and GC3) showing GC contents of 34.16%–45.68%, 31.9%–42.34%, and 29.84%–42.28%, respectively (**Supplementary Table 1**).

Each of the 37 chloroplast genomes was annotated with 130–139 functional genes, including 84–89 protein-coding genes (PCGs), 37 transfer RNA (tRNA) genes, and 8 ribosomal RNA (rRNA) genes (**Supplementary Table 1**). Based on the different functions of the genes, these genes were classified into protein synthesis and DNA replication genes (71–78), photosynthesis-related genes (44–46), other protein-coding genes (6), and genes of unknown function (6–9) (**Supplementary Table 3**). Among the 130–139 functional genes, 15–17 genes contained a single intron, while 3 genes (*clpP1*, *ycf3*, and *rps12*) contained 2 introns.

### 3.2 Analysis of simple sequence repeats and dispersed repeats

This study analyzed the simple sequence repeats (SSRs) and dispersed repeat sequences in the chloroplast genomes of 37 species from 7 genera of the subtribe Leptoboeinae.

**Figure 3:**
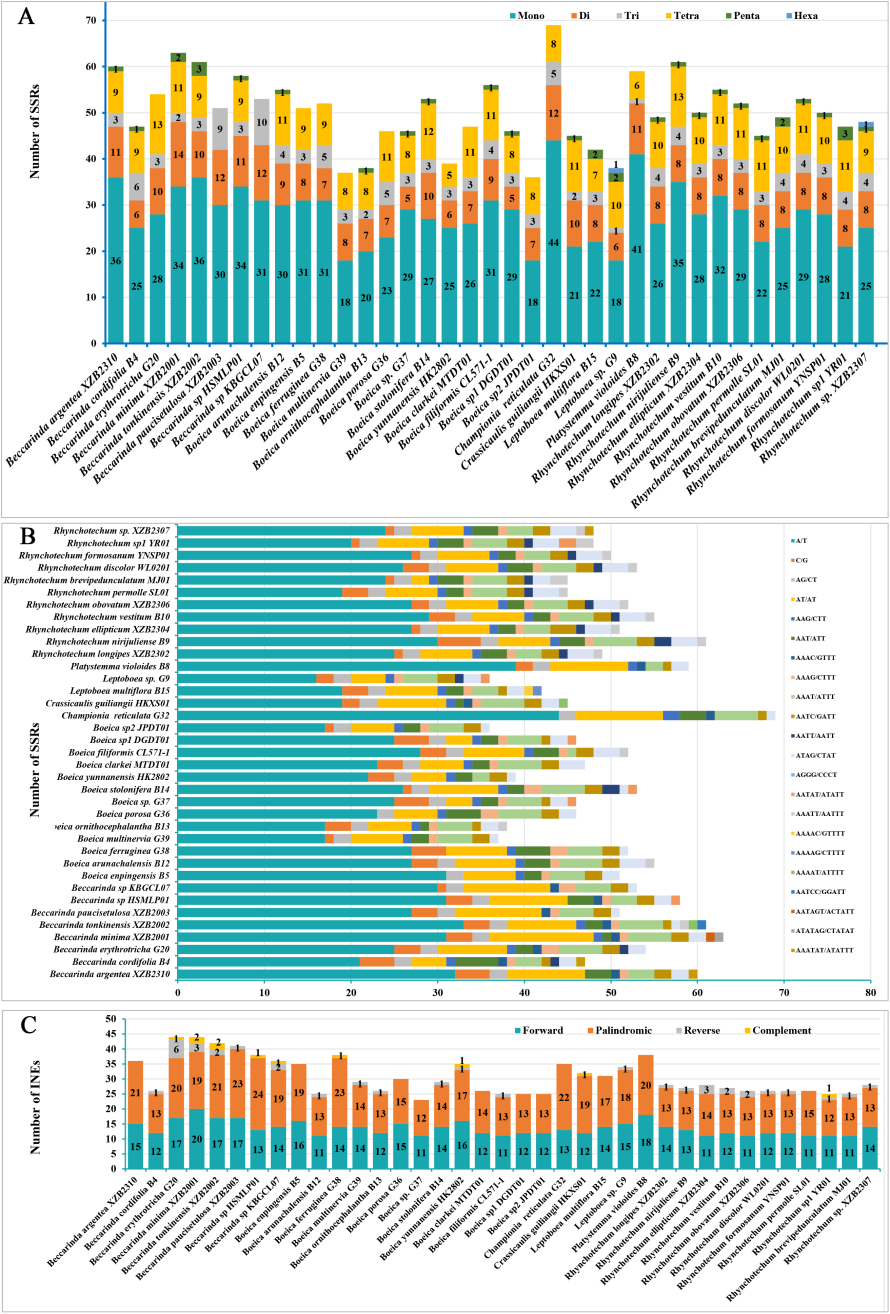
Comparative analysis of repeat sequences in 37 chloroplast genomes of the subtribe Leptoboeinae. **(A)** Number of 6 types of SSRs; **(B)** Number of SSRs with different repeat motifs; **(C)** Dispersed repeats.

A total of 1861 SSRs were detected (**Supplementary Table 4**), with each species containing 37 *Boeica multinervia* K. Y. Pan (G39) to 69 *Championia reticulata* Gardner (G32) SSRs. These included 18–44 mononucleotide (Mono) repeats, 5–14 dinucleotide (Di) repeats, 1–9 trinucleotide (Tri) repeats, 0–13 tetranucleotide (Tetra) repeats, 0–3 pentanucleotide (Penta) repeats, and 0–1 hexanucleotide (Hexa) repeats (**Figure 3A**). The repeat units were mainly composed of adenine (A) and thymine (T), with mononucleotide A/T repeats being the most abundant, followed by dinucleotide AT/AT repeats (**Figure 3B**). Among the chloroplast genomes of the 37 species, only *Leptoboea* sp. G9 and *Rhynchotechum* sp. XZB2307 contained all 6 types of SSRs (**Figure 3A**).

Dispersed repeats are distributed throughout the genome and include four types: forward (F), reverse (R), palindromic (P), and complement (C) repeats (Jelinek et al., 1980). In this study, a total of 1179 dispersed repeats were detected across the 37 chloroplast genomes (**Supplementary Table 4**). The number of dispersed repeats per species ranged from 23 in *Boeica* sp. G37 to 44 in *Beccarinda erythrotricha* G20 and *Beccarinda minima* XZB2001. These included 11–20 forward repeats, 12–23 palindromic repeats, 0–6 reverse repeats, and 0–2 complement repeats. Forward and palindromic repeats were the most abundant types, and reverse and complement repeats were absent in some species (**Figure 3C**).

### 3.3 Expansion and contraction of SC/IR boundaries

**Figure 4:**
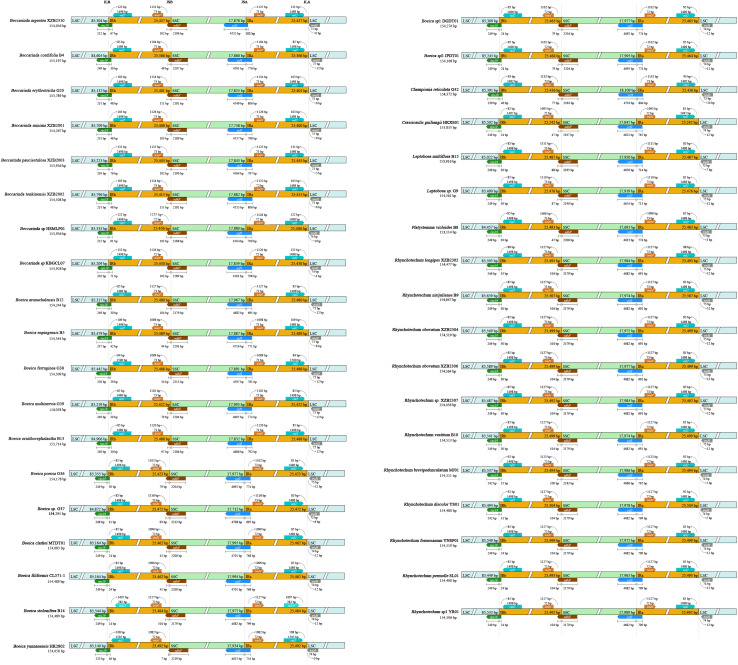
Comparison of SC/IR boundaries among 37 chloroplast genomes of the subtribe Leptoboeinae.

The 37 chloroplast genomes of the subtribe Leptoboeinae exhibit a typical quadripartite structure, with 4 junctions between the single-copy (SC) regions and the inverted repeat (IR) regions. The results showed that the SC/IR boundaries of the 37 chloroplast genomes are relatively conserved. The LSC/IRB boundary (JLB line) is located in the *rps19* gene, the IRB/SSC boundary (JSB line) lies within the overlapping region of the *ndhF* gene and the pseudogene *ycf1*, the SSC/IRA boundary (JSA line) is located in the *ycf1* gene, and the IRA/LSC boundary (JLA line) falls in the intergenic region between *rpl2* and *trnH* (**Figure 4**).

At the LSC/IRb boundary, the *rps19* gene spans the JLB line, with a length of 202–250 bp in the LSC region and 24–76 bp in the IRb region. At the IRb/SSC boundary, the *ndhF* gene crosses the JSB line, with a length of 7–111 bp in the IRb region and 2,161–2,219 bp in the SSC region. In some species, the *ndhF* gene shows partial extension into the IRb region, such as in *Boeica arunachalensis* D. Borah, R. Kr. Singh, Taram & A. P. Das (B12), *B. ornithocephalantha* F.Wen, T.V.Do & Y.G.Wei (B13), and *B. stolonifera* K. Y. Pan (B14), which exhibit additional expansions of 25 bp, 18 bp, and 14 bp, respectively. At the SSC/IRa boundary, the *ycf1* gene spans the JSA line, with a length of 4,549–4,716 bp in the SSC region and 102–806 bp in the IRa region. At the IRa/LSC boundary, the *rpl2* gene is located to the left of the JLA line, 84–1,607 bp away, while *trnH* is positioned to the right of the JLA line, at a distance of 0–14 bp.

### 3.4 Comparative analysis of whole chloroplast genome alignment and nucleotide polymorphism

**Figure 5:**
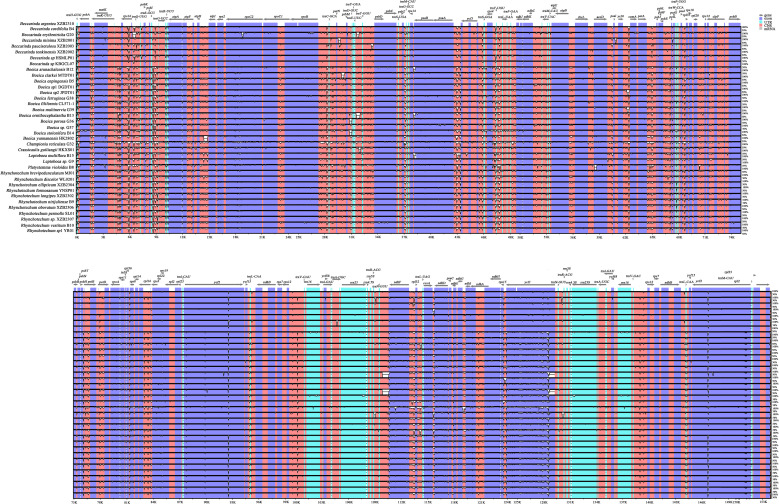
Global alignment analysis of 37 chloroplast genomes of the subtribe Leptoboeinae using the LAGAN algorithm on the mVISTA platform, with *Beccarinda argentea* XZB2310 as the reference sequence.

Using *Beccarinda argentea* (Anthony) Burtt (XZB2310) as the reference sequence, multiple whole-genome alignments of the chloroplast genomes from 37 species of the subtribe Leptoboeinae were performed and visualized (**Figure 5**). The results showed that the SC regions exhibited higher nucleotide variability than the IR regions, and the non-coding regions were more variable than the coding regions. Within the coding regions, all genes were relatively conserved except for *ycf1*.

**Figure 6:**
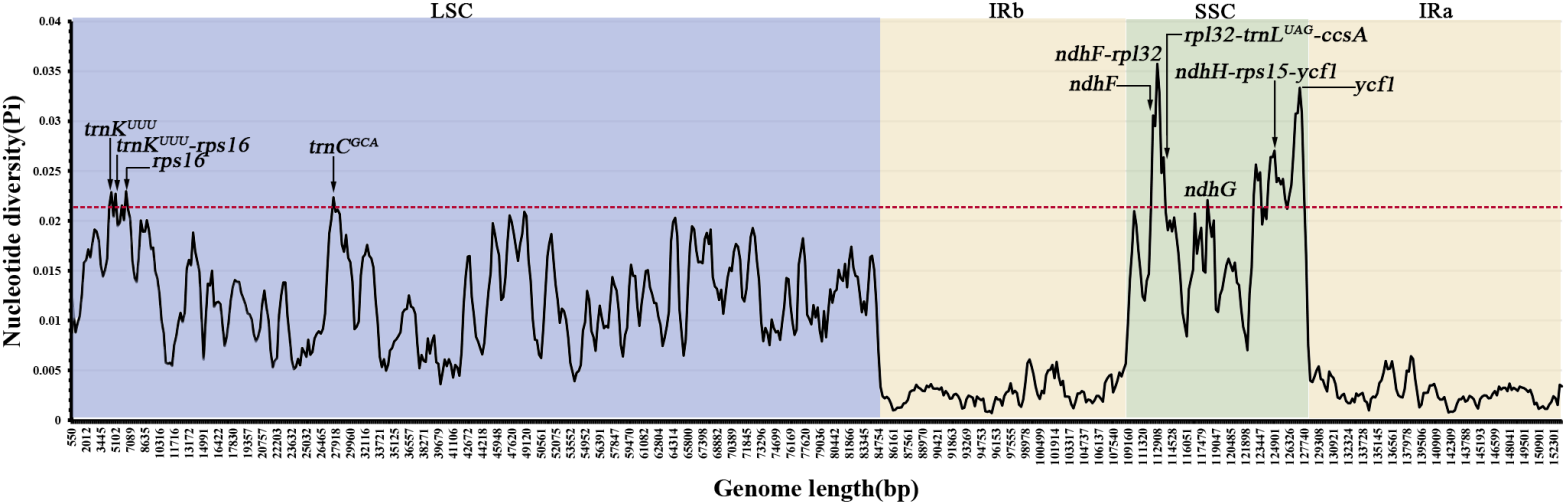
Comparison of nucleotide diversity (Pi) among 37 chloroplast genomes. The red dashed line represents the lowest Pi value within the top 5% of windows, and regions with Pi values above this line are identified as highly variable regions.

Comparative analysis of nucleotide polymorphism (Pi values) in 800 bp sliding windows across the alignment of the 37 chloroplast genomes showed that Pi values ranged from 0 to 0.03574, with the lowest Pi value among the top 5% windows being 0.02136. A total of ten mutation hotspot regions (Pi > 0.02136) were identified as potential molecular markers: *trnK^UUU^*, *trnK^UUU^*–*rps16*, *rps16*, *trnC^GCA^*, *ndhF*, *ndhF*–*rpl32*, *rpl32*–*trnL^UAG^*–*ccsA*, ndhG, *ndhH–rps15–ycf1* and *ycf1*. All ten mutation hotspots were located in the SC regions, whereas Pi values in the IR regions were generally lower and more conserved (**Figure 6**).

### 3.5 Codon usage bias analysis

In this study, CDS longer than 300 bp were selected, resulting in a total of 58 CDS used for codon usage bias analysis. A total of 64 codons were detected, with the total number of codons ranging from 19,558 *Beccarinda cordifolia* (Anthony) B. L. Burtt (B4) to 21,391 *Championia reticulata* G32. UGA (stop codon) and AUU (encoding isoleucine) were the least and most frequently used codons among the 37 chloroplast genomes, respectively, while cysteine (Cys, C) and leucine (Leu, L) were the least and most abundant amino acids, respectively (**Supplementary Table 5**).

**Figure 7:**
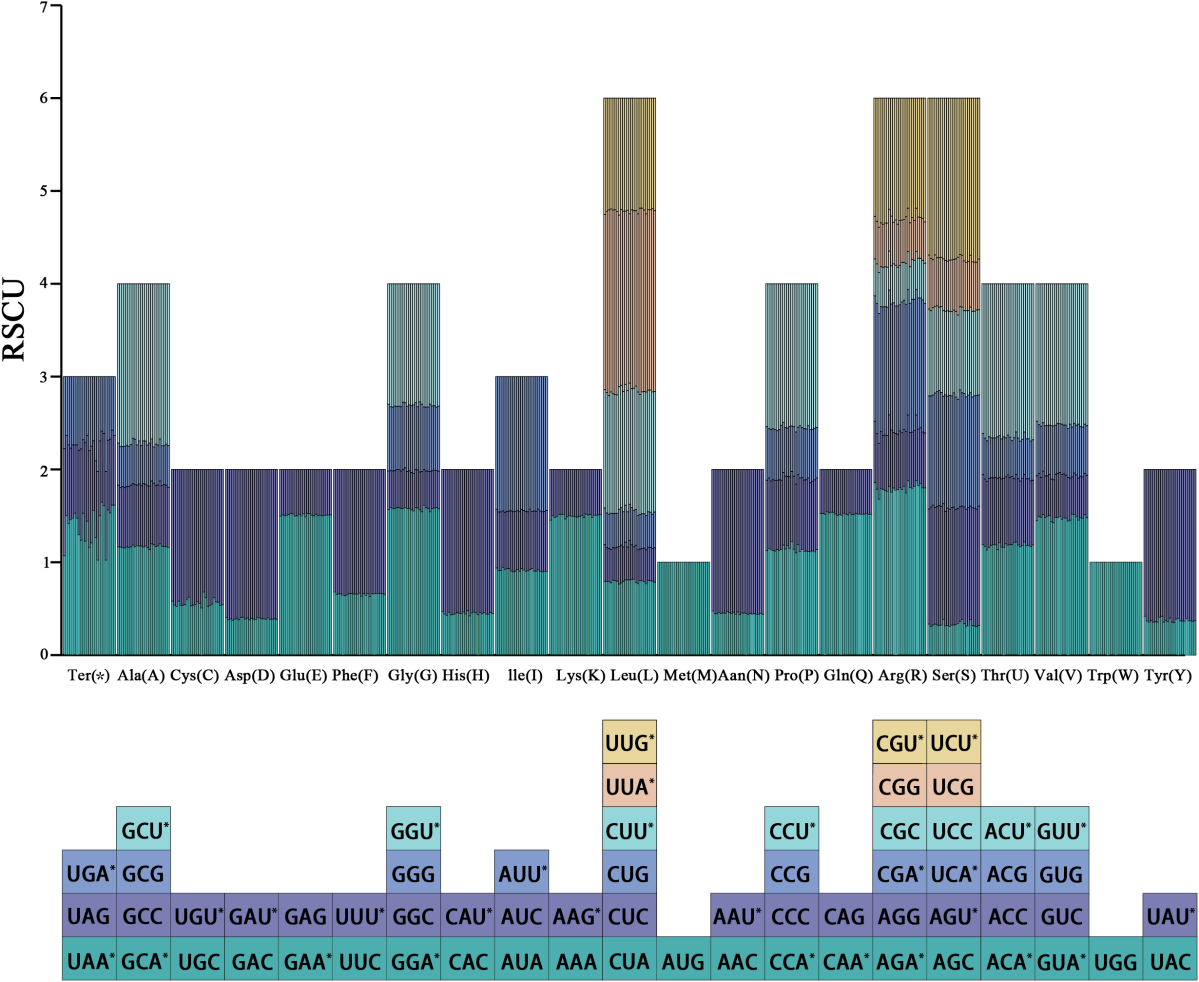
Relative synonymous codon usage (RSCU) values of codons encoding 20 amino acids and stop codons in the 37 chloroplast genomes. Each column in the bar chart represents one species, and color in the column graph corresponds to the codons depicted below the figure. “*” indicates codons with RSCU > 1.

Analysis of codon RSCU values for the 37 chloroplast genomes showed (**Figure 7**) that among the 64 codons, 31 codons had RSCU > 1 and were considered high-frequency codons, 29 of which ended with A/U. Conversely, 31 codons had RSCU < 1 and were considered low-frequency codons, 28 of which ended with G/C. Methionine (Met, M) and tryptophan (Trp, W) are each encoded by a single codon, AUG and UGG respectively, with RSCU = 1, showing no codon usage preference.

### 3.6 Phylogenetic analysis

In this study, we constructed phylogenetic trees for all genera of the subtribe Leptoboeinae using four datasets (concatenated CDSs, coalescent CDSs, complete chloroplast genome sequences, and nrDNA). The plastid tree derived from the concatenated CDSs showed a topology similar to the coalescent tree but conflicted with the nrDNA tree. Most major clades received strong support across all datasets, consistently indicating that *Championia* is the sister group to the remaining members of the Trichosporeae (BS = 100; PP = 1; LPP = 1). After excluding *Championia*, Leptoboeinae was recovered as monophyletic (BS = 100; PP = 1; LPP = 1) (**Figure 1**; **Supplementary Figure 2**). Within the subtribe, ten major clades were resolved: Clade B (*Platystemma*), Clade C (*Crassicaulis*), Clade D (*Beccarinda*), five clades within *Boeica* (E1–E5), Clade F (*Leptoboea*), and Clade G (*Rhynchotechum*). In both the plastid and coalescent trees, Clade E1 formed a sister relationship with the combined clade E2+E3+E4+E5+F+G. Clade E2 was sister to Clade F. Clades E3 and E4 each formed independent lineages. Additionally, Clade E5 was nested within Clade G, rendering *Rhynchotechum* paraphyletic. These results further confirm that *Boeica* is non-monophyletic.

**Figure 8:**
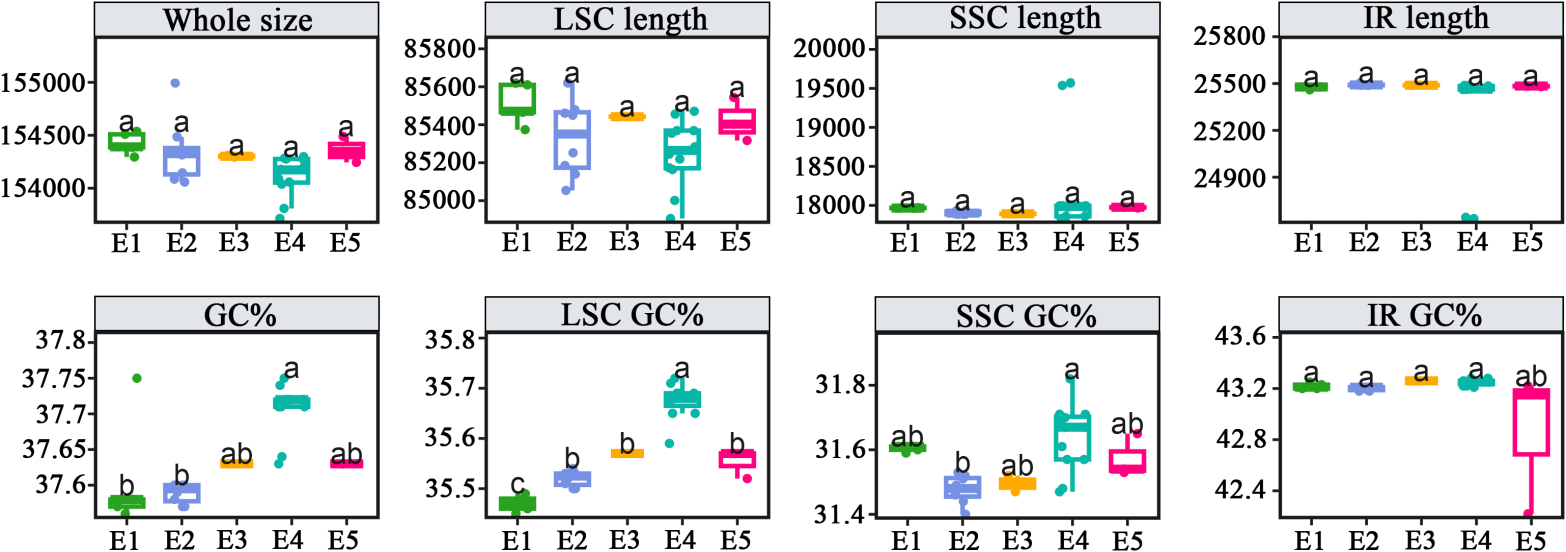
Comparative analysis of genomic characteristics among clades within *Boeica*. The x-axis represents the clade numbers, and different lowercase letters indicate significant differences among the five clades at the P < 0.05 level.

All chloroplast genomes of Leptoboeinae exhibit the conserved quadripartite structure. The genome sizes, the lengths of the LSC, SSC, and IR regions, and their corresponding GC contents for each genus are shown in **Supplementary Figure 3** and **Supplementary Table 1**, and the one-way ANOVA results are provided in **Supplementary Table 6**. The statistical analyses indicate that the ten clades show significant differences in chloroplast genome size and GC content, and the subclades of *Boeica* also exhibit significant variation in GC content (**Figure 8**).

## 4 Discussion

### 4.1 Conservatism of the chloroplast genomic structure in the subtribe Leptoboeinae

In this study, the chloroplast genomes of 37 species of the subtribe Leptoboeinae were all found to possess the typical quadripartite structure. Their chloroplast genome organization, gene types, gene content, SC/IR boundaries, and codon usage bias are relatively conserved. The high degree of conservation of chloroplast genomes has also been observed in the tribe Trichosporeae, as well as in the genera *Primulina* and *Paraboea* (Clarke) Ridley within Gesneriaceae (Cui et al., 2023; Gu et al., 2024; Wang et al., 2022), indicating that chloroplast genome structure across Gesneriaceae is subject to strong evolutionary and functional constraints. The sizes of the chloroplast genomes ranged from 153,114 bp to 154,647 bp, which falls within the general size range (115 kb–165 kb) of photosynthetic plant chloroplast genomes (Jansen et al., 2005; Zhang et al., 2021). This narrow size range further supports the hypothesis that plastome evolution in Leptoboeinae has been dominated by purifying selection rather than by large-scale structural rearrangements. Given that most species of this subtribe inhabit shaded understory environments with relatively stable microclimatic conditions, strong functional constraints associated with photosynthetic efficiency may have limited structural divergence of chloroplast genomes (Kan et al., 2024). Similar ecological filtering effects have been proposed to maintain plastome stability in other shade-adapted angiosperm lineages (Magota et al., 2024). The GC content of the chloroplast genome is unevenly distributed, with the highest GC content in the IR regions and the lowest in the SSC region, which may be related to the presence of 4 rRNA genes (rrn4.5, rrn5, rrn16, rrn23) in the IR regions (Lan et al., 2022; Peng et al., 2022; Yan et al., 2022). In addition, we found that the GC content range of Leptoboeinae species is consistent with that previously reported for other chloroplast genomes of Gesneriaceae, such as Trichosporeae (37.2%–37.8%; Cui et al., 2023) and *Primulina* (37.71%–38.80%; Gu et al., 2024). This consistency suggests that base composition evolution of the chloroplast genome has remained relatively conserved during the diversification of Leptoboeinae, providing a stable molecular background for phylogenetic inference.

Contraction, expansion, or gene loss of the IR regions often leads to changes in the length of the genome sequence (Goulding et al., 1996; Raubeson et al., 2007; Wang et al., 2008). In this study, the SC/IR boundaries of the 37 chloroplast genomes were relatively conserved, with no obvious IR expansion or contraction observed, which is largely consistent with the SC/IR boundary patterns reported in other species of Gesneriaceae (Cui et al., 2023; Gu et al., 2024). Although the boundary regions of chloroplast genomes are relatively stable, expansions and contractions of the inverted repeat (IR) regions can lead to the extension of the *ndhF* gene located at the boundary, a phenomenon that has also been observed in other plant lineages (Asaf et al., 2021; Liang et al., 2022), and are more likely attributable to minor boundary shifts rather than directional evolution of the inverted repeat regions. This further supports the overall structural stability of chloroplast genomes in the subtribe Leptoboeinae.

Codon usage bias is crucial for understanding species evolution, predicting gene function, and estimating expression levels (Uddin et al., 2015). Analysis of codon usage bias showed that the frequencies of 64 codons were generally similar across the 37 chloroplast genomes. A total of 31 high-frequency codons, 33 low-frequency codons, and 2 unbiased codons were identified. Among the high-frequency codons, most ended with A/U, except for UUG (encoding leucine). In contrast, the low-frequency codons tended to end with C/G. This pattern is consistent with the relatively low GC3 content (**Supplementary Table 1**) and is also in line with previous findings (Hsieh et al., 2022; Cui et al., 2023; Gu et al., 2024).

### 4.2 Repeats in the chloroplast genome

In the chloroplast genomes of the 37 species, the detected SSRs were mainly composed of mono-, di-, tri-, and tetranucleotide repeats. The SSR repeat units were predominantly composed of A or T, with A/T and AT/AT repeats being the most common, while other types were relatively rare. This pattern is consistent with the major SSR repeat units identified in the chloroplast genomes of other Gesneriaceae species (Hsieh et al., 2022; Cui et al., 2023; Gu et al., 2024). Previous studies have shown that among all SSR types, the contents of A and T bases are much higher than those of G and C, which corresponds to the general SSR characteristics of angiosperm chloroplast genomes (Asaf et al., 2020). This pronounced AT bias is consistent with the overall low GC content of chloroplast genomes and further reflects the presence of biased mutational pressure that favors replication slippage in AT-rich regions (Massouh et al., 2016; Kan et al., 2024). In addition, forward and palindromic repeats were the main types of dispersed repeats, which is consistent with reports in other chloroplast genomes (Yang et al., 2023b; Qin et al., 2025). The dominance of forward and palindromic dispersed repeats further suggests that even in the absence of large-scale genome rearrangements, localized recombination events or slipped-strand mispairing may drive small-scale variations in chloroplast genomes (Wang and Lanfear, 2019; Cauz-Santos, 2025).

### 4.3 Identification of highly variable regions as DNA barcodes

In this study, results from multiple alignments of the 37 chloroplast genomes and mVista similarity analyses showed that the IR regions are more conserved than the SC regions, with greater nucleotide divergence observed in the non-coding regions of the SC and in the *ycf1* gene. This pattern is largely attributable to the homogenizing effect of concerted evolution between inverted repeat sequences, which reduces sequence divergence within the IR regions (Ruhlman et al., 2017). Further quantification of nucleotide polymorphism levels identified a total of ten mutational hotspot regions, providing important insights into the evolutionary dynamics of chloroplast genomes in the subtribe Leptoboeinae. Among these regions, *ycf1*, *trnK^UUU^*–*rps16*, and *ndhF* are considered potential efficient molecular markers, which have also been reported in other angiosperms (Wang et al, 2024b), indicating that these loci represent evolutionarily unstable regions. Their elevated variability is likely attributable to relaxed functional constraints or higher nucleotide substitution rates (Dong et al., 2015; Dugas et al., 2015). Moreover, the candidate DNA barcodes identified in this study establish a practical bridge between species identification and deeper phylogenetic reconstruction, and thus have direct implications for taxonomic studies, conservation genetics, and evolutionary biology within Gesneriaceae.

### 4.4 Phylogenetic relationships

#### 4.4.1 Phylogenetic relationships of genera within the subtribe Leptoboeinae

Based on CDS sequences, this study reconstructed the most comprehensive phylogeny to date for the subtribe Leptoboeinae, including all 37 sampled species representing all genera, using both coalescent and concatenation approaches. The two methods produced largely congruent topologies at the generic level (**Figure 1**; **Supplementary Figure 2**), indicating that chloroplast genome data provide reliable phylogenetic signals for resolving deep relationships within the subtribe. However, differences in the placement of several species are likely attributable to chloroplast-specific features and methodological biases (Wu et al., 2015). Recent work has demonstrated the presence of cytonuclear conflict within the subtribe (Yang et al., 2025). Compared with Yang et al., our coalescent tree differs in several respects: *Platystemma* is recovered as basal; *Boeica yunnanensis* group with *B. enpingensis*; *B. ferruginea* forms an independent lineage; and *Boeica filiformis* is nested within *Rhynchotechum*. In contrast, Yang et al. recovered *Crassicaulis* as basal; *Boeica yunnanensis* as a separate lineage; *B. ferruginea* and *B. enpingensis* as sister taxa; and *Boeica filiformis* as sister to *Rhynchotechum*. Such topological incongruence may result from hybridization (Robertson et al., 2010), chloroplast capture (Yang et al., 2021), introgression (Pelser et al., 2010; Cai et al., 2021), or incomplete lineage sorting (ILS) (Feng et al., 2022). Future work integrating broader nuclear genomic data will be necessary to resolve these reticulate evolutionary patterns. Overall, confirming the monophyly of Leptoboeinae supporting the exclusion of *Championia* from Leptoboeinae, consistent with earlier hypotheses (Middleton et al., 2015; Ranasinghe et al., 2024). In this study, species of *Boeica* were scattered across five clades and showed varying degrees of cyto-nuclear conflict. However, the type species of *Boeica* (*Boeica fulva*) was not sampled here or in any previous phylogenetic work, leaving its placement among the recovered clades (E1–E5) unresolved. Traditional morphological characters used to delimit genera within Leptoboeinae show partial correspondence with the phylogenetic relationships reconstructed here. For example, *Platystemma* and *Beccarinda* are closely related, sharing the traits of free sepals and ovoid ovaries; however, *Platystemma* is distinguished from other Leptoboeinae genera by having only 1–2 terminal leaves, while *Beccarinda* is set apart by nearly transverse capsule insertion (Möller et al., 2017). The relationships among *Boeica*, *Leptoboea*, and *Rhynchotechum* have long been established (Fritsch, 1894; Wei et al., 2010), likely due to their shared morphological traits of being suffruticose with stems and having four stamens (Weber et al., 2013). In contrast, *Leptoboea* and *Boeica* bear capsules, whereas *Rhynchotechum* produces indehiscent berries; additionally, *Leptoboea* has opposite leaves and anthers that do not fuse apically. *Crassicaulis* is morphologically distinct from other genera by its stout stems and the presence of glandular hairs on the pedicel, calyx, and corolla (Yang et al., 2025). The polyphyly of *Boeica* has been repeatedly recognized in previous studies, but neither morphological nor molecular phylogenetic analyses have fully resolved it (Yang et al., 2020; 2023a; Yang et al., 2025). Morphologically, leaf arrangement and other characters are often unstable and overlap with those of related genera; molecularly, pervasive cyto-nuclear conflict and signals of reticulate evolution further complicate phylogenetic inference. Within the clades defined here (E1–E5), among which E1 and E3 can be distinguished from the other *Boeica* lineages by their nearly actinomorphic corollas and opposite leaves (Wang et al., 1990, 1998). whereas no distinct morphological characters were found for E2, E4 and E5. This pattern suggests that a combination of morphological stasis or convergence and reticulate evolution has blurred lineage boundaries within *Boeica*.

#### 4.4.2 The polyphyly of *Boeica* may be partially congruent with its chloroplast genome structure

Structural variation in chloroplast genomes often contributes to the formation of genetic diversity among lineages, which is not only associated with phylogenetic relationships but also linked to environmental adaptation (Šmarda et al., 2014). The polyphyly of *Boeica* has been repeatedly demonstrated in previous studies, yet its taxonomic controversy remains unresolved (Ranasinghe et al., 2024; Yang et al., 2025). In this study, we found that clades E1 and E3 of *Boeica* can be distinguished by morphological characters, whereas clades E2, E4, and E5 are difficult to separate. Structural variation in chloroplast genome has proven valuable for reconstructing evolutionary relationships (Li et al., 2015; Zhan et al., 2024), and this is also supported by our findings (**Figure 8**). Analyses of chloroplast genome structure and ANOVA indicate that genome size and GC content vary among clades, largely driven by significant differences in LSC length. Although clade E2 is closely related to E3 and E4, their GC contents differ markedly, particularly in the LSC, likely due to the region’s high gene density, functional diversity, and rapid evolutionary rate (Li et al., 2020; Qin et al., 2025; Yu et al., 2025). Notably, clade E5 differs significantly from clades E2, E3, and E4. Habitat analyses show that E5 occurs mainly in shaded tropical forests, and elevated GC content has been associated with adaptation to extreme temperatures (Carels et al., 1998; Šmarda et al., 2014), suggesting that environmental stress may drive GC-content evolution in this clade. Although E5 is morphologically distinct from *Rhynchotechum*, its nested position within that genus and its GC-content differences relative to the G clade of *Rhynchotechum* further highlight incongruence, a pattern also observed in other plant groups (Keating et al., 2023; Oyston et al., 2022). Because multiple *Boeica* clades have overlapping distributions, plastid genomic divergence may reflect independent lineage evolution. Overall, chloroplast genome characteristics provide new evidence for resolving the polyphyly of *Boeica*, a framework successfully applied to other polyphyletic groups (Zhang et al., 2023; Zhan et al., 2024). While taxonomic problems remain unresolved, this study offers new directions for future evolutionary and systematic research, and integrating morphological character evolution may further clarify generic boundaries.

## 5 Conclusion

This study presents the first comprehensive chloroplast genome analysis of the subtribe Leptoboeinae and successfully assembled the chloroplast genomes of 17 species within the group. Comparative analyses revealed that the overall genome structure, gene categories, gene content, and codon usage bias of these chloroplast genomes are relatively conserved. Across the 37 chloroplast genomes, with a total of 1,861 SSRs and 1,179 dispersed repeats identified. Ten hypervariable regions were detected, which represent potential molecular markers for genus-level delimitation. Codon usage patterns were highly similar, with the same 31 codons most frequently used. Phylogenetic analyses based on chloroplast genomes recovered well-resolved phylogenetic trees. Although conflicts were detected between the plastid tree and the coalescent tree regarding the phylogenetic placement of some taxa, both trees consistently supported the polyphyly of *Boeica*, which was resolved into five distinct lineages, and revealed that *Rhynchotechum* is paraphyletic as a consequence. The monophyly of *Beccarinda* was confirmed, whereas the boundaries of *Rhynchotechum* and *Boeica* require redefinition. In addition, structural variation within chloroplast genomes partially explains the polyphyly of *Boeica*. Significant differences were observed in GC content, LSC GC content, and SSC GC content among closely related lineages. Overall, these findings offer new insights for future evolutionary studies and systematic classification, and highlight the need for further integration of nuclear genomic data with morphological evidence to resolve the complex polyphyly of *Boeica*.

## Data Availability

The datasets presented in this study can be found in online repositories. The names of the repository/repositories and accession number(s) can be found in the article/**Supplementary Material**.

## Supplementary material

Supplementary Table 1 Chloroplast genome features of 37 species of the tribe Leptoboeinae.

Supplementary Table 2 Sampling information of 37 species of the tribe Leptoboeinae.

Supplementary Table 4 Number of SSRs and INEs in 37 chloroplast genomes.

Supplementary Table 6 Results of one-way ANOVA for chloroplast genome features among different clades of the tribe Leptoboeinae.
