# Supplementary material for: Comparative and phylogenetic analysis of chloroplast genomes in the subtribe Leptoboeinae (Gesneriaceae)
Source: Front Plant Sci. 2026 Mar 23;17:1766257. doi: 10.3389/fpls.2026.1766257 (PMC13050898; doi:10.3389/fpls.2026.1766257)
Supplement: Supplementary Table 1 — Chloroplast genome features of 37 species of the tribe Leptoboeinae. [file DataSheet1.zip › Supplementary Material Presentation/Supplementary Figure S1.docx]

**Comparative and Phylogenetic Analysis of Chloroplast Genomes in the Subtribe Leptoboeinae (Gesneriaceae)**





**Supplementary Figure S1.** Gene map of the subtribe Leptoboeinae
