# Supplementary material for: Comparative and phylogenetic analysis of chloroplast genomes in the subtribe Leptoboeinae (Gesneriaceae)
Source: Front Plant Sci. 2026 Mar 23;17:1766257. doi: 10.3389/fpls.2026.1766257 (PMC13050898; doi:10.3389/fpls.2026.1766257)
Supplement: Supplementary Table 1 — Chloroplast genome features of 37 species of the tribe Leptoboeinae. [file DataSheet1.zip › Supplementary Material Presentation/Supplementary Figure S2.docx]

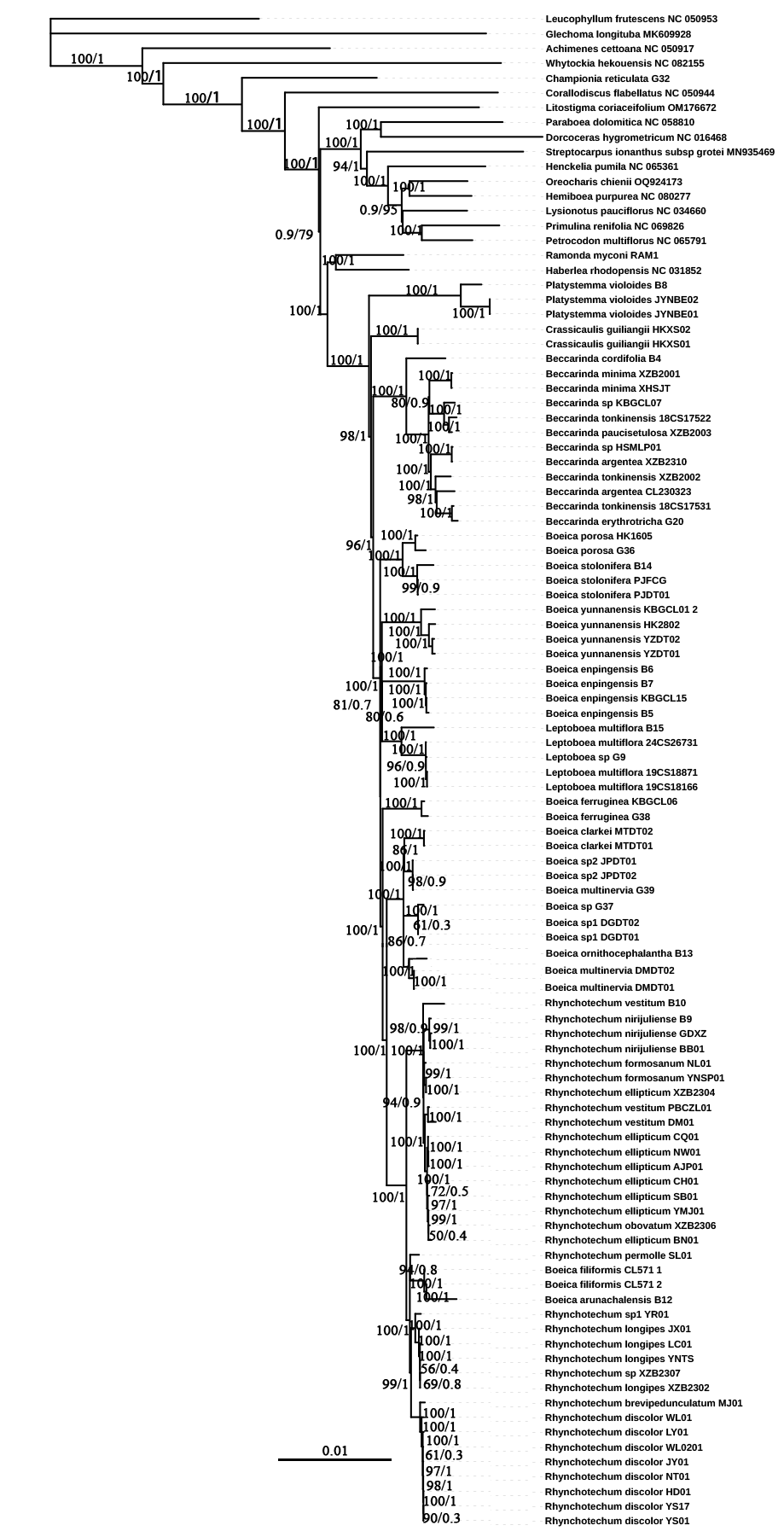


Figure 1. Phylogenetic relationships of the tribe Leptoboeinae inferred from 81 CDSs using the concatenation method. The BI and ML trees are combined, with Bayesian posterior probabilities (PP) and maximum likelihood bootstrap support values (BS) shown on the branches.


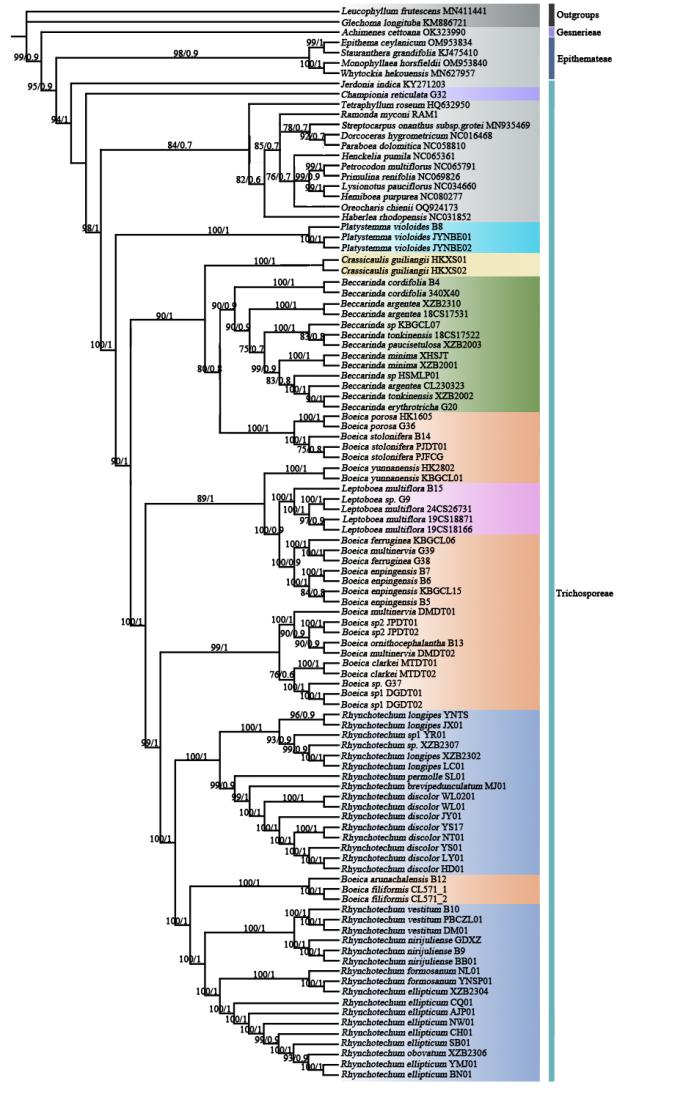


Figure 2. Phylogenetic tree based on nuclear ribosomal DNA sequences: combined presentation of the BI tree and ML tree, with branch values indicating Bayesian inference (BI) posterior probabilities (≥0.5) and maximum likelihood (ML) bootstrap support values (≥70%).


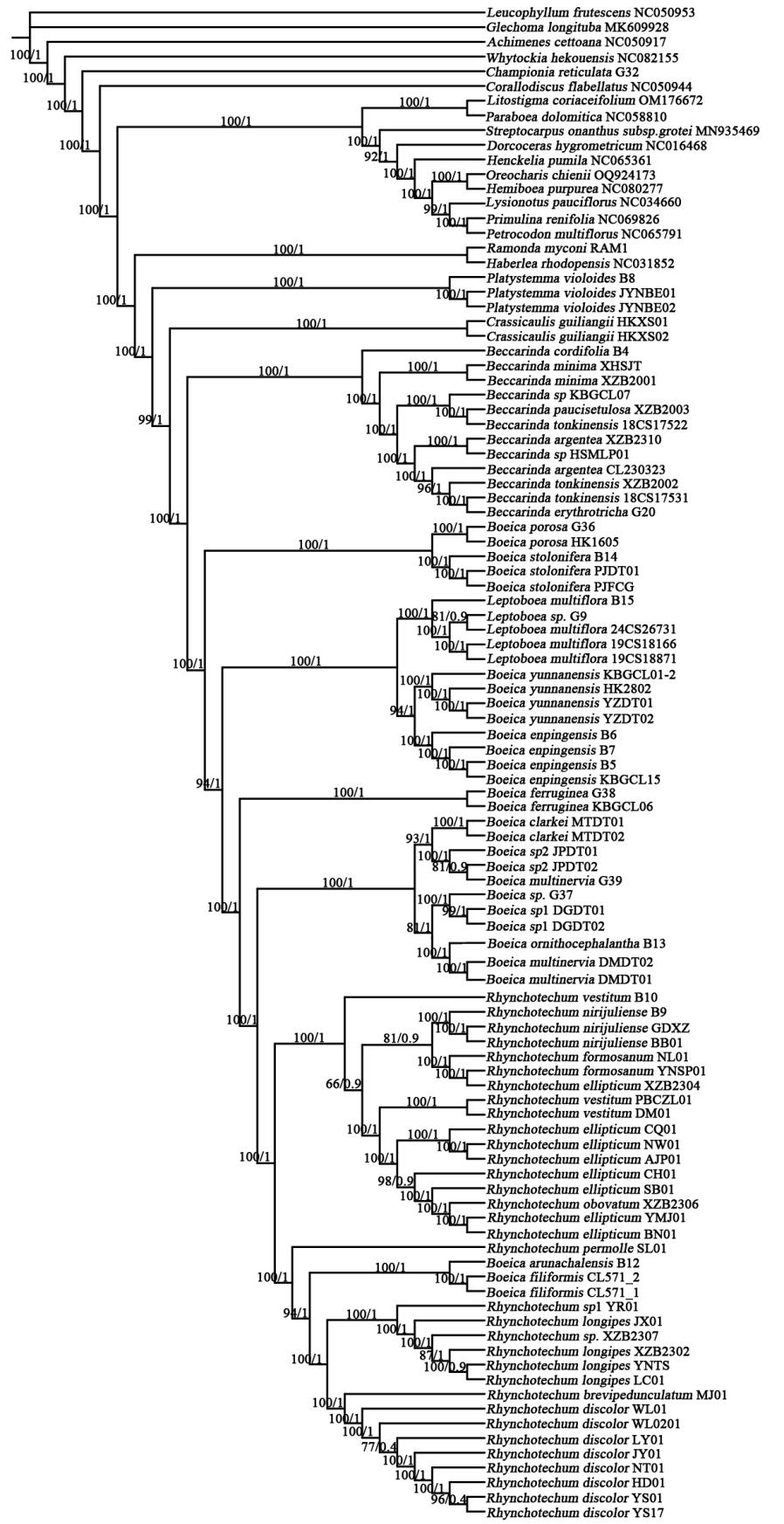


Figure 3. Phylogenetic relationships of the tribe Leptoboeinae inferred from complete chloroplast genome sequences (with one IR region removed) using the concatenation method. The BI and ML trees are combined, with Bayesian posterior probabilities (PP) and maximum likelihood bootstrap support values (BS) shown on the branches.
