# Supplementary material for: Comparative and phylogenetic analysis of chloroplast genomes in the subtribe Leptoboeinae (Gesneriaceae)
Source: Front Plant Sci. 2026 Mar 23;17:1766257. doi: 10.3389/fpls.2026.1766257 (PMC13050898; doi:10.3389/fpls.2026.1766257)
Supplement: Supplementary Table 1 — Chloroplast genome features of 37 species of the tribe Leptoboeinae. [file DataSheet1.zip › Supplementary Material Presentation/Supplementary Figure S3.docx]

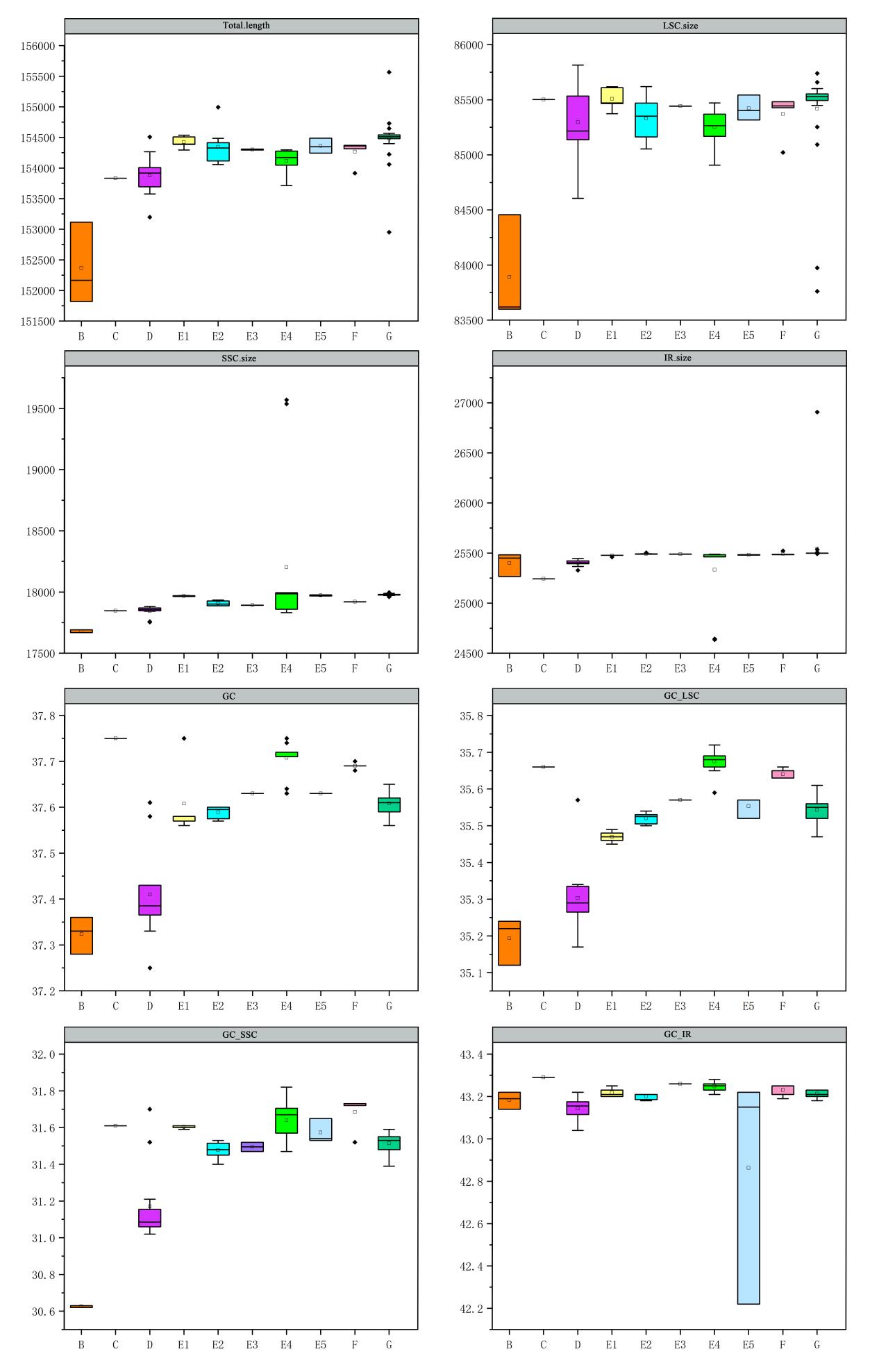


Figure S3. Boxplots showing the comparison of chloroplast genome features among different clades within Leptoboeinae (Black dots indicate outliers). The x-axis corresponds to the clade numbers, and the results of the one-way ANOVA are summarized in Table 3.
